# CD19-Negative Acute Lymphoblastic Leukemia (ALL): A Case Report and Review of Literature on a Rare Phenomenon De Novo and a Future Induced Struggle in Relapse

**DOI:** 10.3390/ijms27073203

**Published:** 2026-04-01

**Authors:** Marta Arrabito, Emanuela Cannata, Piera Samperi, Manuela La Rosa, Luca Lo Nigro

**Affiliations:** 1 Center of Pediatric Hematology Oncology, Azienda Policlinico, San Marco, 95123 Catania, Italy; marta-arrabito@hotmail.it (M.A.); e.cannata80@gmaul.com (E.C.); psamperi@policlinico.unict.it (P.S.); 2 Cytogenetic-Cytofluorimetric-Molecular Biology Lab, Center of Pediatric Hematology Oncology, Azienda Policlinico, San Marco, 95123 Catania, Italy; larosa_manuela@libero.it; 3 XLI Cycle of PhD Program in Translational Biomedicine, University of Catania, 95100 Catania, Italy

**Keywords:** acute lymphoblastic leukemia, B-lineage, CD19 negative, hypercalcemia

## Abstract

Acute lymphoblastic leukemia (ALL) is the most common childhood malignancy, with most cases arising from B-cell precursors expressing the CD19 marker. CD19 negativity in B-lineage ALL (B-ALL) is very rare de novo and poses diagnostic and therapeutic challenges. Sometimes, de novo CD19-negative B-ALL is associated with hypercalcemia, which is a potentially life-threatening metabolic disorder in children, rarely occurring in cancers. Most often it is reported in solid tumors, and few cases are reported in pediatric acute leukemia. CD19-negative B-ALL relapse is also an increasing dramatic event, secondary to immunotherapy. We describe a ten-month-old infant presenting with hypercalcemia, anemia, and osteolytic bone lesions. Bone marrow analysis revealed CD10-positive and CD19-negative B-ALL. The patient achieved complete remission but later experienced two relapses and died of respiratory failure after a second allogeneic hematopoietic stem cell transplantation (HSCT). Only nine cases of de novo CD19-negative B-ALL have been reported so far. Many are associated with hypercalcemia and osteolytic lesions. However, here we highlight the clinical impact of the more common secondary form of CD19-negative B-ALL as a relapse of CD19-positive ALL, right after the administration of targeted immunotherapy.

## 1. Introduction

Acute lymphoblastic leukemia (ALL) is the most common pediatric malignancy and accounts for approximately 25% of childhood cancers [1]. The first classification of ALL relied on the morphological criteria of the French–American–British (FAB) classification. But since the introduction of immunophenotype analysis with flow cytometry (FCM), diagnosis of leukemia as being of the B- or T-lineage has largely been done using lineage markers that are expressed on the surfaces of cells or at the intracytoplasmic level.

ALL mostly originates from B-cell precursors that express the pan B-cell marker CD19 in almost all cases [2]. This marker acts as a “gating” antigen in the immunophenotyping analysis of leukemic cells. The *CD19* gene is located on the short arm of chromosome 16 (16p11.2), and its expression on blasts has been shown to be the most predictable marker for assignment to the B-lineage. De novo CD19-negative B-ALL is quite uncommon, and very few cases have been described [3].

Some cases of ALL are associated with hypercalcemia at the onset of the disease. Hypercalcemia is a rare and potentially life-threatening metabolic disorder in children that is defined as a serum calcium level of more than 12 mg/dL. The differential diagnosis involves metabolic, nutritional, drug-induced, genetic, inflammatory, and neoplastic aspects. Although common in adults (20–30% of cases), hypercalcemia is rare in pediatric cancers, and its overall incidence ranges between 0.4 and 1.3% [4]. Most often, hypercalcemia is reported in cases of solid tumors, such as Ewing’s sarcoma, neuroblastoma, rhabdomyosarcoma, and non-Hodgkin lymphoma. A few cases have also been described in the context of acute leukemia.

We report a case of an infant who presented with CD19-negative B-cell precursor (BCP)-ALL associated with hypercalcemia, as well as a review of the relevant literature. Based on this de novo case, we highlight the phenomenon of CD19-negative B-ALL, which was observed as a relapse after CD19-directed T-cell immunotherapies. This is an increasing and challenging event that should be considered in future protocols. For this reason, we also report a review of relapsing cases, which are increasingly being described in comparison to de novo cases. We would emphasize the uniqueness and innovative role of our case description.

## 2. De Novo CD19-Negative B-ALL: A Case Description

A 10-month-old child was admitted for vomiting, irritability, weight loss, loss of appetite, dehydration, and low-grade fever that had occurred in the last 2 weeks. He also presented dysmorphic features such as low-set ears and a broad forehead, as well as delayed psychomotor development and vertebral malformations with a butterfly appearance in Th1, Th8, Th10, and Th11. Blood tests showed microcytic anemia (hemoglobin (Hb) 7 g/dL, mean corpuscular volume (MCV) 57 fl), platelets and white cells in normal ranges, hypercalcemia (18 mg/dL), and decreased parathyroid hormone values.

**Figure 1:**
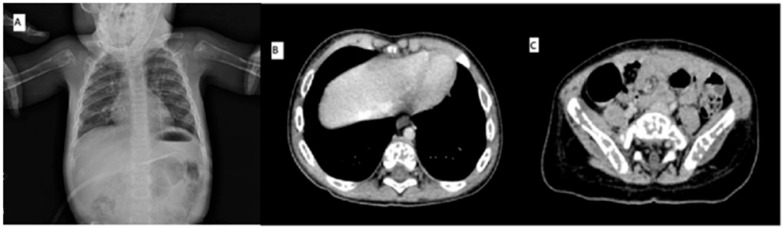
Skeletal X-ray (**A**) and total-body CT scan (**B**,**C**) showing widespread osteolytic areas. (**A**) Skeletal X-ray showing diffuse osteolytic lesions involving ribs and long bones; (**B**) total-body CT scan with contrast detecting multiple osteolytic areas in the axial and appendicular skeleton, particularly affecting thoracic vertebrae and pelvic bones.

**Figure 2:**
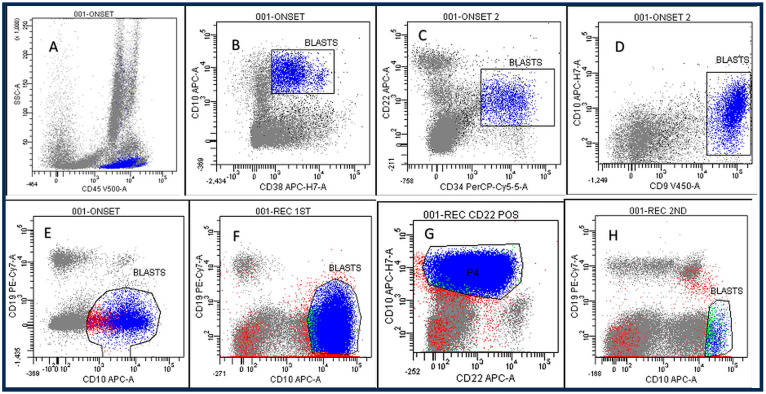
Flow cytometry (FCM) analyses performed at the onset of the disease (**A**–**E**), at the first relapse (**F**,**G**) and at the second relapse (**H**). (**A**–**E**) FCM showed an expanded population (24%) of blasts expressing CD45 dim, CD10, CD22, CD38, and CD9 but not CD19 antigens; (**B**,**C**) FCM at the first relapse demonstrated recurrence of the original leukemic clone, with CD19-negative blasts (55%) expressing CD10 and CD22, with increased intensity (**D**) FCM at the second relapse revealed a persistent leukemic population (2.5%) with the same immunophenotypic features. All these FCM findings were consistent with molecular analyses.

Skeletal X-rays and total-body computed tomography (CT) were performed and showed widespread osteolytic areas (Figure 1). Rehydration therapy and furosemide were administered, which led to a slight reduction in serum calcium levels, although they remained above the normal limit, and the symptoms persisted. Because of the hypercalcemia, anemia, and osteolytic lesions, the child underwent bone marrow aspiration (BMA). FCM analysis indicated that 24% of lymphoblasts were compatible with a diagnosis of B-lineage CD10 ^pos^/CD19 ^neg^ ALL (Figure 2A). Blasts were identified using a stepwise gating strategy. First, cells were gated on CD45 expression and side scatter (SSC) to define the blast population (CD45 dim/low, low SSC). Subsequently the gated population was analyzed for CD10 and CD19 expression. The leukemic cells consistently showed CD10 positivity and absence of CD19 expression, supporting the definition of a CD19-negative leukemic population. The only CD19-positive cells detected through FCM were the residual mature B-lymphocytes. Appropriate controls and fluorescence thresholds were used to define CD19 negativity. In such cases, blast identification relies on CD45/SSC gating and the integration of additional B-lineage markers, according to established flow cytometry guidelines as previously published [5,6]. Central nervous system involvement was excluded by lumbar puncture.

Cytogenetic analysis indicated a normal karyotype, and negative results were obtained in molecular tests using polymerase chain reaction (PCR) methods to search for the *KMT2A/MLL* fusion partner (Table 1). In accordance with the patient’s age (<1 year), he was subjected to the Interfant-06 international protocol [7]. The induction phase led to complete morphological remission, but not molecular remission (Marker 1. IgH VH3 JH4: 3.3 × 10^−3^; consolidation and reinduction). After 2 days of prednisone treatment, a rapid improvement in clinical conditions and normalization of calcium values occurred (from 18 to 8.9 mg%).

A bone marrow evaluation on day 15 indicated 5% blasts in FCM, which meant that the state of the disease was considered as low risk. During the maintenance phase, 17 months after the onset of ALL, the patient had leg pain and refused to walk, so BMA was performed, and an isolated early relapse of B-lineage CD10 ^pos^/CD19 ^neg^ ALL was diagnosed (Figure 2B,C). Thus, therapy was started according to the second-line protocol IntReALL-2010 SR-SIB with the addition of Berlin–Frankfurt–Münster (BFM)-like blocks (HR1–HR2–HR3).

A family donor with identical human leukocyte antigens (HLAs) was available (a sister), so the child underwent allogeneic bone marrow hematopoietic stem cell transplantation (allo-HSCT) after conditioning with Busulfan–Fludarabine–Thiotepa, as indicated by the Interfant-06 Protocol. At the first check-up at one month after the HSCT, the child showed adequate engraftment with 100% donor chimerism. A bone marrow examination was performed 5 months after the HSCT, and FCM and molecular analyses showed the presence of 2.5% CD10 ^pos^/CD19 ^neg^ blast cells with MRD-IgH > 5 × 10^−2^, which confirmed that a second relapse of B-lineage ALL had occurred (Figure 2D).

Based on the CD19 negativity and the CD22 positivity, we treated the child with two cycles of anti-CD22 monoclonal immunotherapy with inotuzumab ozogamicin (InO), and a second round of allo-HSCT was performed using total-body irradiation (TBI) conditioning. We administered the first cycle of InO as suggested by the phase-II study [8]. BMA analysis confirmed that morphological and cytofluorimetric remission had occurred. We then applied another as the patient was a “good responder” [8].

**Figure 3:**
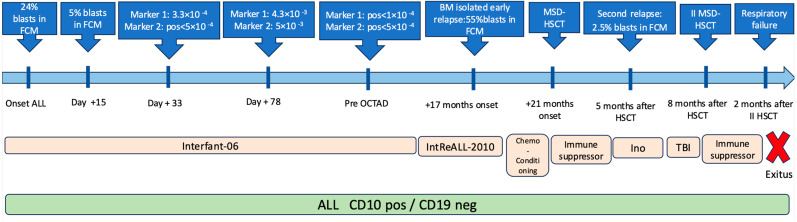
Timeline illustrating FCM, molecular analyses and administered therapies according to the time points and events of the disease.

Next, the child underwent the second HSCT program. After 2 months, the patient was admitted to our ward with a general state of malaise characterized by a loss of appetite, constipation, drowsiness, and a suspected pulmonary infection. Due to worsening general conditions, the child was admitted to intensive care but died a few days later due to respiratory failure (Figure 3).

## 3. De Novo CD19-Negative B-ALL in Children: A Review of the Literature

CD19 is a B-cell-specific marker that is expressed in all stages of B-lymphocytes, including plasma cells. FCM diagnosis and monitoring of MRD in B-ALL is mainly established in the CD19-based primary gating strategy, and it is a challenge to study B-ALL without CD19 expression.

De novo CD19-negative B-ALL is a very rare event, and few cases have been mentioned in the literature.

Ghodke et al. [9] reported three cases of CD19-negative B-ALL, of whom two were children: a 2-year-old male and a 10-year-old female. The results of cytogenetic analyses were negative for recurrent translocations [9]. Moon et al. [10] described a case of a 6-year-old girl with CD45-negative and CD19-negative B-ALL presenting blasts with an atypical morphology and variable size, a high nuclear cytoplasmic ratio, and distinct nucleoli. Amplification of the *MLL* gene led to a positive result in fluorescent in situ hybridization (FISH) [10]. Sultan et al. presented a case of a 9-month-old infant with CD19-negative B-ALL who presented with hypercalcemia and lytic bone lesions at diagnosis [11].

Hypercalcemia is a rare complication of childhood cancers. A large retrospective study from St. Jude Children’s Research Hospital examined a period of 29 years and involved 2816 patients with lymphohematopoietic malignancy. Only 10 children presented ALL-induced hypercalcemia, and seven of them had hypercalcemia at diagnosis [12]. Hypercalcemia is often observed in children with specific features such as older age (10–20 years), a white blood cell count of less than 20,000/mm^3^ at diagnosis with few or absent circulating blasts according to peripheral blood smear, and aberrant expression of myeloid antigens such as CD13 and CD33 [13]. There are also some published cases that describe B-ALL and hypercalcemia associated with the translocation t(17; 19) [14].

In contrast, our patient was an infant with B-ALL lacking the t(17; 19) translocation and the expression of CD13/CD33 antigens. However, he did not show the presence of circulating blasts (aleukemic presentation) in peripheral blood and had a normal blood count. Furthermore, in this case, hypercalcemia was associated with several osteolytic lesions, and there is little evidence in the literature about this type of presentation. Dhivyasree et al. reported a case of a 4-year-old girl with B-ALL who presented with severe hypercalcemia and radiological evidence of osteolytic lesions [15]. Lokadasan et al. and Khayyam et al. respectively described a 15-year-old boy and a 3-year-old boy with ALL presenting with hypercalcemia, disseminated lytic bone lesions, normal blood counts, and an absence of circulating blasts [16,17].

Only nine cases of CD19-negative B-ALL have been described in the literature, and most of them showed osteolytic lesions and hypercalcemia (Table 2). Interestingly, an absence of circulating blasts in the peripheral blood and primary prevalence in pediatric patients have been common features among all published cases, as in our patient. Niizuma et al. [18] reported two children with precursor CD19-negative B-ALL who had marked hypercalcemia, disseminated osteolysis, and considerably elevated serum levels of tumor necrosis factor-alpha (TNF-alpha) and IL-6. These findings imply that increased osteoclastic bone resorption stimulated by TNF-alpha and IL-6 may cause parathormone-related protein (PTHrP)-independent hypercalcemia in a few patients with precursor B-ALL lacking CD19 expression [18]. Tahara et al. proved that the receptor activator of NF-κB ligand (RANKL), which is expressed on the surface of bone marrow stromal cells such as osteoblasts, links to the receptor activator of NF-κB (RANK) on the osteoclast precursors, leading to the differentiation and activation of osteoclasts and causing hypercalcemia [19]. This mechanism is related exclusively to de novo CD19-negative B-ALL. The CD19-negative ALL relapses after immunotherapy are caused by other factors as mentioned below.

In some cases, this condition could be related to genetic alterations.

Hussein et al. [20] performed single-nucleotide polymorphism (SNP) array analysis for two patients with hypercalcemia and CD19-negative BCP-ALL. Both children were infants (one year of age), and one of them had a deletion of *TBL1XR1* that had been described in *ETV6/RUNX1*-positive B-ALL [20]. We planned to perform an SNP array analysis for our patient as well, which was also related to the dysmorphic features and delayed psychomotor development, but the child died before the test could be performed.

## 4. Review of Relapse of CD19-Negative ALL in Children

CD19-negative B-ALL is more often observed as a relapse after CD19-directed T-cell immunotherapies like blinatumomab or chimeric antigen receptor (CAR)-T-cells, which occurs through the loss of the target molecule due to the selective pressure of the targeting drugs. This mechanism is one of the keyways in which leukemia cells evade immunotherapy. Locatelli et al. [21] showed that CD19-negative relapse more commonly follows CAR-T-cell therapy than blinatumomab (30% vs. 22.5%). The median durations of follow-up were 29.3 and 20.4 months for blinatumomab and CAR-T-cell therapies, respectively [21]. Mejstríková et al. reported four pediatric patients who experienced CD19-negative B-ALL relapse after blinatumomab treatment, and one of these cases presented with a *KMT2A/MLL* rearrangement [22]. This genetic alteration is often associated with lineage switch, which represents another phenomenon of escape induced by the selective pressure of lineage-specific therapies, as described recently [23]. Different mechanisms have been suggested to contribute to leukemic lineage switch such as the roles of bipotential progenitors, cell reprogramming or de-differentiation as a consequence of stem cell plasticity [24,25].

Rabilloud et al. [26] described a pediatric patient who developed a CD19-negative relapse after CD19 CAR-T-cell therapy. Using a single-cell RNA sequencing (scRNAseq) approach, they showed that CD19-negative leukemic cells were present before CAR-T-cell therapy, which means that the relapse resulted from the selection of this rare clone [26]. The outgrowth of pre-existing CD19-negative clones is one of the proposed mechanisms for CD19-negative relapse after immunotherapy, including protein alterations by mutations in the CD19 gene or alternative messenger RNA splicing. Sotillo et al. [27] showed de novo frameshift and missense mutations in CD19-exon 2 in CD19-negative relapse samples. They also detected spliced CD19 mRNA species, identifying SRSF3 as a splicing factor involved in exon 2 retention, which induces expression of the N-terminally truncated CD19 variant in relapsed B-ALL [27]. Also, Fischer et al. described an alternatively spliced CD19 mRNA isoform lacking exon 2 but manifested in leukemic blasts at diagnosis, demonstrating that some of the CD19 isoforms pre-exist at diagnosis [28]. Furthermore, Orlando et al. identified genetic mutations in CD19 at the time of CD19-negative relapse that could lead to a truncated protein with a nonfunctional or absent transmembrane domain and, thereby, to the lack of surface antigen [29].

In some cases, CD22 may be a helpful marker to monitor minimal residual disease (MRD), as well as a therapeutic target. Kamitori et al. [30] described a case of 5-year-old boy with B-lineage ALL who had a second bone marrow relapse and showed leukemic blasts that were negative for CD19 but positive for CD22 after CAR-T-cell therapy. They decided to administer InO monotherapy as a bridge to subsequent allo-HSCT, and the patient remained in complete remission [30].

Aertgeerts et al. [31] reported four children who relapsed after receiving CAR-T cells (tisagenlecleucel) and were treated with InO. Three of them relapsed with CD19 ^neg^/CD22 ^pos^ B-lineage ALL. After the first InO cycle, all achieved complete remission, and after another two or three InO cycles, they underwent allo-HSCT. One patient developed an isolated extramedullary relapse, received palliative radiotherapy, and was in complete remission as of the last follow-up 25 months later. The other patients were also in complete remission at the last follow-up (mean 31.3 months) [31].

Myers et al. [32] examined 22 patients who all presented with highly refractory B-ALL after CD19-directed immunotherapy to test a novel anti-CD22/4-1BB CAR-T-cell construct, CART22-65s. There were 19 patients who were infused (pediatric, *n* = 17; adult, *n* = 2) and 14 (74%) who achieved complete remission with a median follow-up of 38 months. In the pediatric cohort, the median age at infusion was 16.1 years, 16/17 (94%) children had CD19-negative relapses after CD19 CAR-T, and 1/17 (6%) had a CD19-negative relapse after receiving blinatumomab [32] (Table 3).

## 5. Conclusions

Although rare, B-ALL can show de novo negative expression of CD19 that may be associated with bone pain, osteolytic lesions, and hypercalcemia. The absence of CD19 expression and circulating blasts should not detract from the possibility of B-ALL diagnosis. A comprehensive antibody panel with additional B-cell markers in immunophenotyping should be used for routine FCM analysis. Furthermore, loss of CD19 surface expression occurs in relapse after immunotherapy treatment, which leads to escape from CAR/blinatumomab-mediated recognition. This phenomenon represents a challenge that should be faced to improve the outcomes for such patients.

## Figures and Tables

**Table 1:** Main clinical, laboratory and biologic features of the patient at the onset of disease.

Age	Ten Months
Sex	Male
Hemoglobin	7 g/dL
White blood count	8.820/mmc
Platelets	222.000/mmc
Symptoms	Vomiting, irritability, weight loss, dehydration, loss of appetite, fever
Clinical features	Low-set ears, broad forehead, delayed psychomotor development and butterfly vertebral malformations
Instrumental exams (X-ray and CT scan)	Widespread osteolytic lesions
Bone marrow aspirate (flow cytometry)	ALL B-lineage CD10 ^pos^/CD19 ^neg^
Cytogenetic analysis	Normal karyotype
Molecular characterization	Negative for the main translocations

This table summarizes the patient’s demographic information, presenting symptoms, clinical findings, imaging results, bone marrow immunophenotype, cytogenetic analysis, and molecular characterization at the time of initial diagnosis.

**Table 2:** De novo CD19-negative ALL, in children: a review of the literature.

	Age	Sex	Hb g/dL	WBC (×10^9^/L)	PLTs (×10^9^/L)	Hypercalcemia and Osteolytic Lesions	BMA (FCM)	FISH	Outcome
Sultan et al. (2004) [11]	9 months	M	9.6	7.98	318	yes	CD19 ^neg^ CD20 ^neg^ CD34 ^neg^	Negative for *ETV6/RUNX1*, *BCR/ABL*, *MLL*-R, t(8; 14)	Alive 16 months after diagnosis
Niizuma et al. (2007) [18]	2 years	M	7.1	5.9	184	yes	CD19 ^neg^ CD20 ^neg^ CD34 ^neg^	Unknown	Relapse 3 years after onset
	2 years	F	11	14.8	200	yes	CD19 ^neg^ CD20 ^neg^	Unknown	Complete remission
Moon et al. (2007) [10]	6 years	F	6.2	0.5	77	unknown	CD19 ^neg^ CD20 ^neg^ CD10 ^neg^	*MLL* amplification	Complete remission
Hussein et al. (2015) [20]	1 year	M	9.6	6.6	334	yes	CD19 ^neg^ CD20 ^neg^ CD34 ^neg^	Negative for *ETV6/RUNX1*, *BCR/ABL*, *MLL*-R, *CDKN2A*, *TCF3/PBX1*	Complete remission
	1 year	F	7.2	13.9	307	yes	CD19 ^neg^ CD20 ^neg^		Complete remission
Ghodke et al. (2017) [9]	38 years	M	9.2	3.9	146	unknown	CD19 ^neg^ CD45 ^neg^	Negative for *ETV6/RUNX1*, *BCR/ABL*, *MLL*-R, *CDKN2A*, *TCF3/PBX1*	Lost to follow-up
	2 years	M	9.6	2.2	23	unknown	CD19 ^neg^ CD34 ^neg^		Complete remission
	10 years	F	8.8	2.2	372	yes	CD19 ^neg^		Complete remission

Hb: hemoglobin; WBC: white blood count; PLTs: platelets; BMA: bone marrow aspirate; FCM: flow cytometry; neg: negative; *MLL*-R: mixed-lineage leukemia gene rearrangement.

**Table 3:** CD19-negative ALL as relapses in children: a review of the literature.

	Number Pts	Immunotherapy Prior CD19 ^neg^ Relapse	Genetic Abnormalities	Relapse Therapy	Outcome	Follow-Up After Relapse
Mejstríková et al. (2017) [22]	4	Blinatumomab	1 with *KMT2A*/*MLL*-R	unknown	2 alive 2 DOD	unknown
Rabilloud et al. (2021) [26]	1	CD19 CART cells	not present	unknown	unknown	unknown
Kamitori et al. (2021) [30]	1	CD19 CART cells	*ETV6-RUNX1* positivity	inotuzumab and allo-HSCT	CR	2 years
Locatelli et al. (2023) [21]	33	Blinatumomab (10) CD19 CART cells (23)	unknown	unknown	CR	29 months (Blina) 20 months (CAR-T cells)
Myers et al. (2025) [32]	17	Blinatumomab (1) CD19 CART cells (16)	unknown	CART22-65s	74% in CR	median follow-up 38 months
Aertgeerts et al. (2025) [31]	4	CD19 CART cells	not present	inotuzumab	CR	median follow-up 30 months
Lo Nigro et al. (2025) [23]	1	Blinatumomab	*KMT2A/MLL-R*	inotuzumab, Gemtuzumab	DOD	n.a.

Pts: patients; n.a: not applicable; CR: complete remission; DOD: dead of disease.

## Data Availability

The original contributions presented in this study are included in the article. Further inquiries can be directed to the corresponding author.

## Abbreviations

ALL: acute lymphoblastic leukemia; FAB classification: French–American–British classification; CT: computed tomography; BMA: bone marrow aspiration; FCM: flow cytometry; PCR: polymerase chain reaction; BFM: Berlin–Frankfurt–Münster; HLA: human leukocyte antigen; allo-HSCT: allogeneic bone marrow hematopoietic stem cell transplantation; TBI: total-body irradiation; TNF-alpha: tumor necrosis factor-alpha; PTHrP: parathormone-related protein; SNP: single-nucleotide polymorphism; CAR: chimeric antigen receptor; scRNAseq: single-cell RNA sequencing; MRD: minimal residual disease; MCV: mean corpuscular volume; BCP: B-cell precursor; Hb: hemoglobin.
